# The transcriptional response to oxidative stress is part of, but not sufficient for, insulin resistance in adipocytes

**DOI:** 10.1038/s41598-018-20104-x

**Published:** 2018-01-29

**Authors:** Rima Chaudhuri, James R. Krycer, Daniel J. Fazakerley, Kelsey H. Fisher-Wellman, Zhiduan Su, Kyle L. Hoehn, Jean Yee Hwa Yang, Zdenka Kuncic, Fatemeh Vafaee, David E. James

**Affiliations:** 1 Charles Perkins Centre, The University of Sydney, Sydney, NSW 2006 Australia; 2 School of Life and Environmental Sciences, The University of Sydney, Sydney, NSW 2006 Australia; 3 Duke Molecular Physiology Institute, Duke University, Durham, NC 27701 USA; 4 Sydney Medical School, The University of Sydney, Sydney, NSW 2006 Australia; 5 School of Biotechnology and Biomolecular Sciences, The University of New South Wales, Sydney, NSW 2052 Australia; 6 School of Mathematics and Statistics, The University of Sydney, Sydney, NSW 2006 Australia; 7 School of Physics and Australian Institute for Nanoscale Science and Technology, The University of Sydney, Sydney, NSW 2006 Australia

## Abstract

Insulin resistance is a major risk factor for metabolic diseases such as Type 2 diabetes. Although the underlying mechanisms of insulin resistance remain elusive, oxidative stress is a unifying driver by which numerous extrinsic signals and cellular stresses trigger insulin resistance. Consequently, we sought to understand the cellular response to oxidative stress and its role in insulin resistance. Using cultured 3T3-L1 adipocytes, we established a model of physiologically-derived oxidative stress by inhibiting the cycling of glutathione and thioredoxin, which induced insulin resistance as measured by impaired insulin-stimulated 2-deoxyglucose uptake. Using time-resolved transcriptomics, we found > 2000 genes differentially-expressed over 24 hours, with specific metabolic and signalling pathways enriched at different times. We explored this coordination using a knowledge-based hierarchical-clustering approach to generate a temporal transcriptional cascade and identify key transcription factors responding to oxidative stress. This response shared many similarities with changes observed in distinct insulin resistance models. However, an anti-oxidant reversed insulin resistance phenotypically but not transcriptionally, implying that the transcriptional response to oxidative stress is insufficient for insulin resistance. This suggests that the primary site by which oxidative stress impairs insulin action occurs post-transcriptionally, warranting a multi-level ‘trans-omic’ approach when studying time-resolved responses to cellular perturbations.

## Introduction

Insulin resistance is a major risk factor for various metabolic diseases, such as type 2 diabetes, cardiovascular disease, and some cancers. Although its underlying mechanisms are elusive, insulin-responsive tissues such as adipose tissue undergo oxidative stress during insulin resistance^1,2^. Indeed, we have previously shown that oxidative stress unifies numerous triggers of insulin resistance in adipocytes and myotubes^3^. These include hyperinsulinaemia, inflammation, and glucocorticoids *in vitro*, as well as nutrient oversupply *in vivo*^3^. Thus, oxidative stress is an etiological component of insulin resistance^4,5^, yet how it impairs insulin action remains elusive.

Oxidative stress arises from the aberrant production or defective scavenging of reactive oxygen or nitrogen species. These species can react with a range of macromolecules – in particular, they can oxidise exposed cysteine residues within proteins^6^, altering signalling and cellular physiology. To protect the cell from oxidative stress, the cell has two major redox buffering pools, governed by glutathione and thioredoxin. Although both thiol antioxidants, they are not redundant, serving to regulate distinct cellular signalling and metabolic pathways^7^. Inhibiting both glutathione^8–10^ and thioredoxin^11,12^ buffering systems has been linked to insulin resistance and metabolic disease. Studying oxidative stress by inhibiting these buffering pools is advantageous over exogenous oxidants (e.g., H_2_O_2_) as it not only avoids experimental artefacts, such as cysteine oxidation by residual (exogenous) H_2_O_2_ during sample processing, but it targets endogenous systems, resulting in a ‘physiological’ origin of oxidant production. Consequently, we sought to establish an oxidative stress model using this approach to understand the role of physiologically-derived oxidative stress in insulin resistance. In particular, does the cellular response to oxidative stress, rather than the oxidative stress itself *per se*, play a role in inducing insulin resistance?

Since end-point experiments provide only a limited snapshot of cellular physiology, we captured temporal dynamics using time-resolved systems biology. We examined the transcriptome as this can be easily quantified on a genome-wide scale, yet represents an integrated response that responds to numerous insults in an adaptive and rapid way^13,14^. Indeed, cells use intricate signalling cascades, whereby stimuli activate several key transcription factors (TFs), which alter the expression of genes essential to the early stages of the response. This includes other TFs, which regulate their own target genes (TGs). This cycle continues, resulting in hundreds of genes being transcriptionally regulated in a coordinated fashion. This is well-studied in the context of cellular differentiation (e.g., adipogenesis^15^), yet there is limited information about the adipocyte transcriptional response to oxidative stress over time.

To study temporal dynamics at the transcriptional level, altered genes are clustered based on their expression patterns over time^16^. This has the potential to be augmented in a biologically-meaningful way using an approach previously applied to phosphoproteomics data^17^, whereby prior knowledge of kinase-substrate interactions determined the optimal clustering of genes. This could be applied to transcriptional data using known (experimentally-validated) TF-TG interactions from public repositories (e.g., ORTI database^18^), which can then be used to identify enriched TFs within the clusters^17^. This relies on the assumption that the TGs for a single TF will be co-regulated and thus have similar expression patterns^17,18^. Overall, this would enable time-series data to be used to generate transcriptional cascades from context-specific TF-TG interactions^18^. Here, we apply this approach to understand the transcriptional responses to oxidative stress.

Overall, we aimed to determine the role of the cellular response to oxidative stress in the development of insulin resistance. To achieve this, we established a model of oxidative stress in adipocytes and quantified the transcriptional response using time-resolved transcriptomics. We used the resulting gene expression patterns and a repository of validated TF-TG interactions^18^ to reconstruct the transcriptional cascade. This presented a picture whereby the activity of many TFs varied over time, leading to a coordinated response at the pathway level. This shared many features with what is observed in insulin resistance. Exploring this further, validation experiments revealed that this transcriptional response is part of, but not sufficient for, insulin resistance in adipocytes.

## Results and Discussion

### Validation of the BCNU/auranofin model

Oxidative stress contributes to adipocyte insulin resistance^3^, yet the transcriptional responses to oxidative stress in this cell-type have not been studied. Thus, in this study we sought to capture the dynamic response to endogenously produced oxidants in 3T3-L1 adipocytes. These cells share many properties with endogenous adipocytes in humans and rodents, most notably a highly robust insulin-responsive glucose transport system. Thus, these cells have been used widely to study insulin action and insulin resistance^3,19^.

We first established models of oxidative stress in cultured 3T3-L1 adipocytes by targeting the intracellular redox buffering pools using two pharmacological inhibitors: auranofin to inhibit thioredoxin reductase, which recycles peroxiredoxins (PRDXs), and 1,3-bis-(2-chloroethyl)-1-nitrosourea (BCNU) to inhibit glutathione reductase, which recycles glutathione. This leads to dimerisation of PRDXs and glutathione, preventing them from scavenging oxidants, which leads to oxidative stress. We treated cells with these inhibitors at concentrations that minimised toxicity, and assessed the dimerisation status of cytosolic PRDX (PRDX2), mitochondrial PRDX (PRDX3), and glutathione.

**Figure 1:**
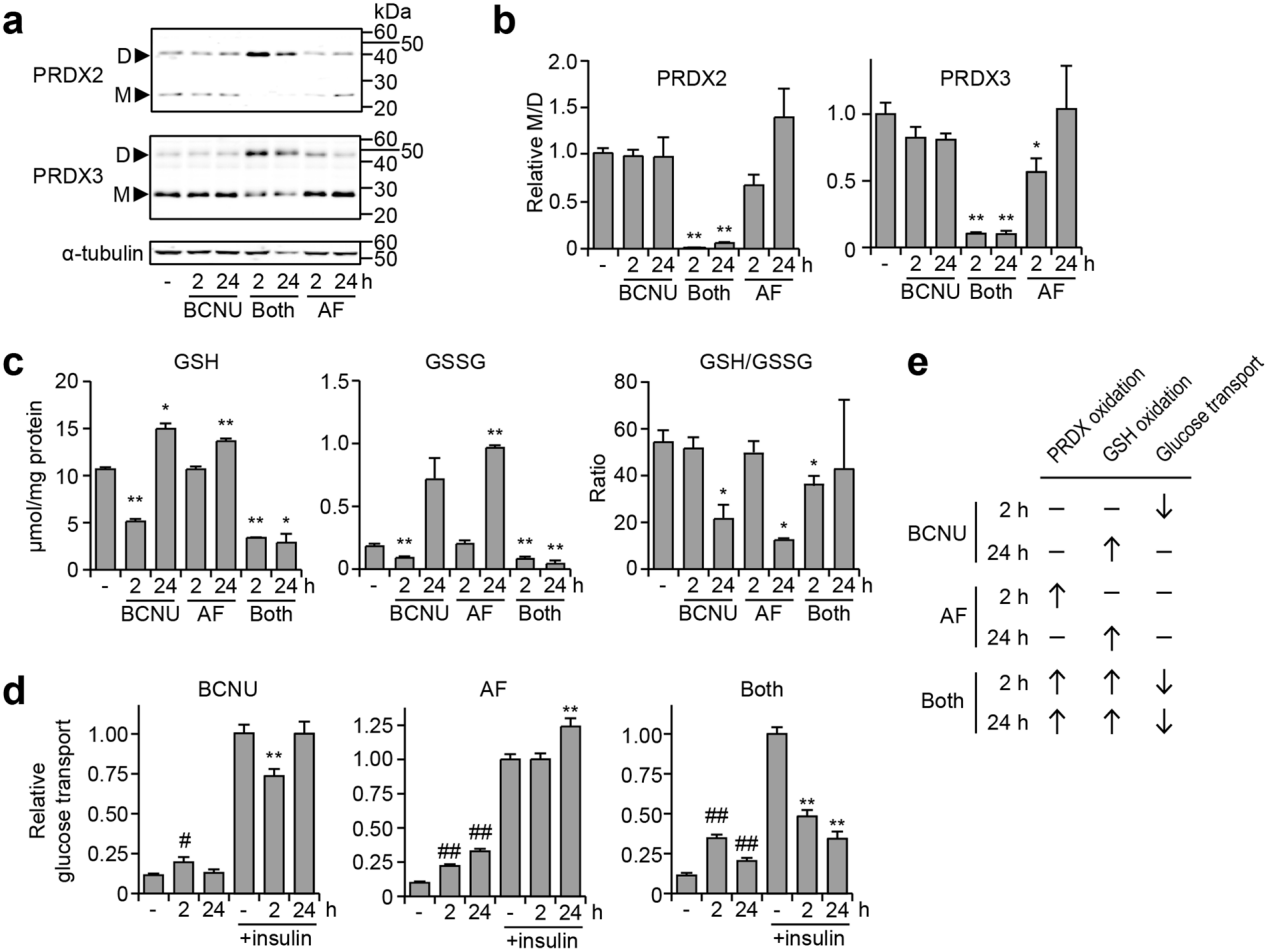
Inhibition of thioredoxin and glutathione recycling induces oxidative stress and insulin resistance in adipocytes. 3T3-L1 adipocytes were treated with 100 µM 1,3-bis-(2-chloroethyl)-1-nitrosourea (BCNU) or 1 µM auranofin (AF) for the indicated time periods. (**a**,**b**) Following treatment, protein was harvested and immunoblotted for the indicated proteins. Full-length blots are presented in Supplementary Figure S1. The intensity of PRDX monomer/dimer was quantified using LI-COR Biosciences Image Studio software in (**b**). (**c**) Following treatment, cells were lysed and assayed for reduced glutathione (GSH) or oxidised glutathione (GSSG). (**d**) Following treatment, cells were assayed for glucose transport. This was performed by using the radiolabelled glucose analog (^3^H-2-deoxyglucose), as described in the Materials and Methods. The uptake of 2-deoxyglucose was normalised to total cellular protein and made relative to the cells treated with insulin alone (without BCNU or auranofin) to obtain ‘relative glucose transport’. (**e**) Summary of the cellular responses to BCNU and AF. All data presented as mean + SEM, from at least n = 3 separate experiments. For (**a**–**c**): *p < 0.05 and **p < 0.01, versus control by two-sample *t*-test. For (**d**): ^#^*p* < 0.05 (versus column 1, no insulin + control); ^##^*p* < 0.01 (versus column 1, no insulin + control); *p < 0.05 (versus column 4, insulin + control); and **p < 0.01 (versus column 4, insulin + control), by two-sample *t*-test. All *p*-values are provided in Supplementary Figure S1.

Individually, the inhibitors had a limited effect on PRDX2 and PRDX3 dimerisation (Fig. 1a–b), yet had a larger effect on the glutathione redox status (Fig. 1c). For instance, auranofin had little effect at 2 h, but by 24 h had significantly increased glutathione dimer (GSSG) and thus lowered the monomer/dimer (GSH/GSSG) ratio (Fig. 1c). This suggests that the glutathione system compensates for loss of thioredoxin reductase activity. BCNU induced a more complex response in glutathione redox status – at 2 h, BCNU lowered both GSH and GSSG leading to a maintained GSH/GSSG ratio, but by 24 h, GSH levels had recovered with a significantly lower GSH/GSSG ratio (Fig. 1c). In contrast to these mild and diverse responses, treatment with both inhibitors simultaneously caused profound oxidation (lower monomer/dimer) of PRDX2 and 3 (Fig. 1a–b) and loss of glutathione at both time points tested (Fig. 1c). The loss of glutathione is likely due to export of GSSG, which has been reported to occur under conditions of severe oxidative stress^20^. Together, these data show that the doses of auranofin and BCNU used in this study elicited mild stress, but the combinatorial inhibition of thioredoxin reductase and glutathione reductase induced a state of oxidative stress.

Next, we examined the impact of BCNU and auranofin on adipocyte insulin sensitivity, which we assayed by measuring insulin-responsive glucose uptake using the glucose analog, 2-deoxyglucose. It has previously been reported that oxidative stress impairs insulin action in adipocytes^3^, so this functional assay provided a measure of whether the disruption of the PRDX and glutathione redox systems had physiological consequences. We found that auranofin alone had little effect and BCNU alone only transiently lowered insulin-responsive glucose uptake (Fig. 1d, left and middle panels), suggesting that individually inhibiting either redox buffering pool is insufficient to cause severe or long-term insulin resistance. However, cells treated with both drugs showed a sustained decrease in insulin responsiveness from 2 to 24 h (Fig. 1d, right panel). Interestingly, these inhibitors also partially increased basal (non-insulin-stimulated) glucose uptake (Fig. 1d, second and third column in each panel), suggesting that oxidative stress uncouples insulin action from glucose transport. Overall, the combination of both drugs caused insulin resistance and provided a model with which to study oxidative stress in adipocytes (Fig. 1e).

### The transcriptional response to BCNU/auranofin overlaps with known oxidative stress markers

**Figure 2:**
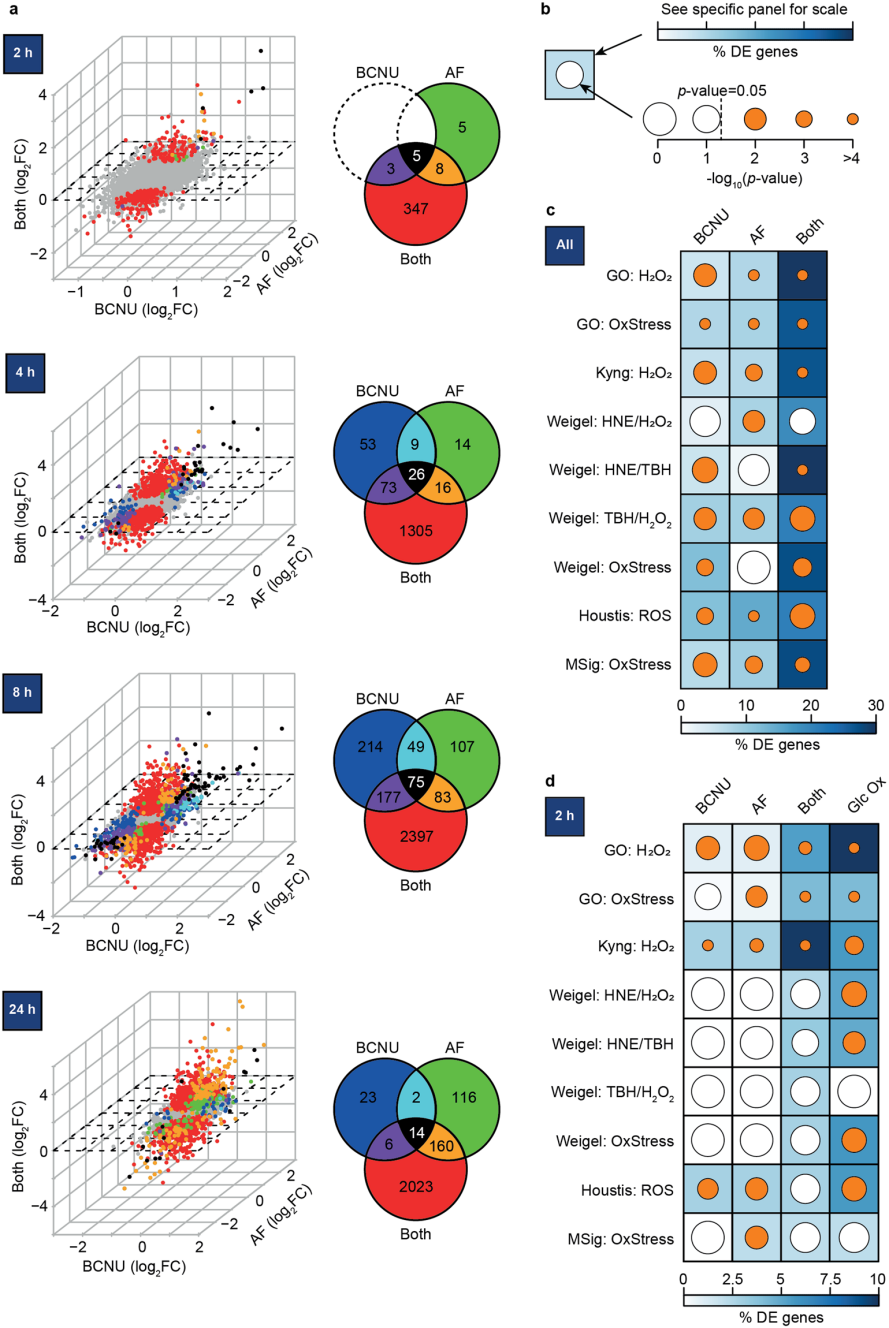
The combined inhibition of thioredoxin and glutathione recycling generates an oxidative stress response in adipocytes. 3T3-L1 adipocytes were treated with 100 µM 1,3-bis-(2-chloroethyl)−1-nitrosourea (BCNU) or 1 µM auranofin (AF) for 2, 4, 8, and 24 h, after which RNA was harvested and subjected to microarray analysis. (**a**) Differentially-expressed genes for each timepoint. The Venn diagram shows the number of overlapping differentially-expressed genes across different drug conditions. In the scatterplots, each axis resembles a condition (BCNU, auranofin, both drugs), depicting fold-change in expression compared to control. Each dot depicts a single gene, with the same colour scheme used in the corresponding Venn diagram. Unaltered genes are depicted in grey. Details can be found in Supplementary Table S1. (**b**–**d**) Comparison of genes in common between oxidative stress gene sets (curated from public databases and literature) and the differentially-expressed genes under each drug condition from (**c**) any time-point or (**d**) only the 2 h time-point. As shown in (**b**), the percentage overlap (relative to the number of genes in each oxidative stress gene set) is reflected by the colour of the background square. The inner circle denotes statistical significance of the overlap by hypergeometric enrichment analysis^65^: smaller circles reflect lower *p*-values, with orange circles denoting *p* < 0.05. Differentially-expressed genes in 3T3-L1 adipocytes treated with glucose oxidase is included as a positive control in (**d**). Details of these analyses can be found in Supplementary Table S2. Abbreviations: GO, Gene Ontology; H_2_O_2_, hydrogen peroxide; HNE, 4-hydroxynonenal; TBH, tert-butylhydroperoxide; OxStress, oxidative stress; ROS, reactive oxygen species.

To observe the dynamic response to oxidative stress, we treated adipocytes with BCNU and/or auranofin and harvested mRNA at multiple time-points up to 24 h. We measured the transcriptome by microarray analysis (Supplementary Table S1). Defining differentially-expressed genes based on fold-change in expression and FDR < 0.05 (as described in the Materials and Methods), we found changes in gene expression as early as 2 h (Fig. 2a). At every time-point, there was a significantly greater transcriptional response when cells received both drugs (*p-*value < 0.0001 when comparing proportions of differentially-expressed genes between drug treatments using Chi-squared test with Yates correction^21^), with 2203 genes differentially-expressed after exposure to both drugs for 24 h. Interestingly, the number of differentially-expressed genes in BCNU-treated cells reduced over time (Fig. 2a, Chi-squared *p*-value < 0.001 when comparing proportions of differentially-expressed genes at each time point compared to adjacent time points), in concordance with the transient decrease in insulin responsiveness (Fig. 1d). Furthermore, when comparing differentially-expressed genes between conditions, the single-drug treatments had substantial overlap with the other conditions, whilst treatment with both drugs generated a significantly larger number of unique differentially-expressed genes (Fig. 2a).

To confirm that these differentially-expressed genes included expected transcriptional responses to oxidative stress, we examined the number of differentially-expressed genes found in published datasets for oxidative stress (Supplementary Table S2). We considered datasets that contained at least 30 genes and as a positive control, we included transcript data from adipocytes incubated with glucose oxidase. This provides a continuous source of exogenous hydrogen peroxide, which we have previously shown to cause insulin resistance in adipocytes^3^. We depicted the overlap of our data with these datasets using two symbols (Fig. 2b): the colour of the square denotes the proportion of oxidative stress genes that were differentially expressed in our data, whilst the inner circle denotes whether this is statistically significant. Thus, smaller circles reflect lower *p*-values, revealing more of the background square’s colour to symbolise a greater overlap of our data with oxidative stress.

Treatment with BCNU and auranofin together resulted in gene expression changes with a greater number of genes implicated in responses to oxidative stress (Fig. 2c). Since the glucose oxidase treatment was for 2 h, we compared the BCNU and auranofin treatments at only 2 h, and found the same conclusion (Fig. 2d). Thus, the co-treatment with BCNU and auranofin generated a transcriptional response that was consistent with the cells experiencing oxidative stress.

### Coordinated transcriptional regulation occurs in response to oxidative stress

**Figure 3:**
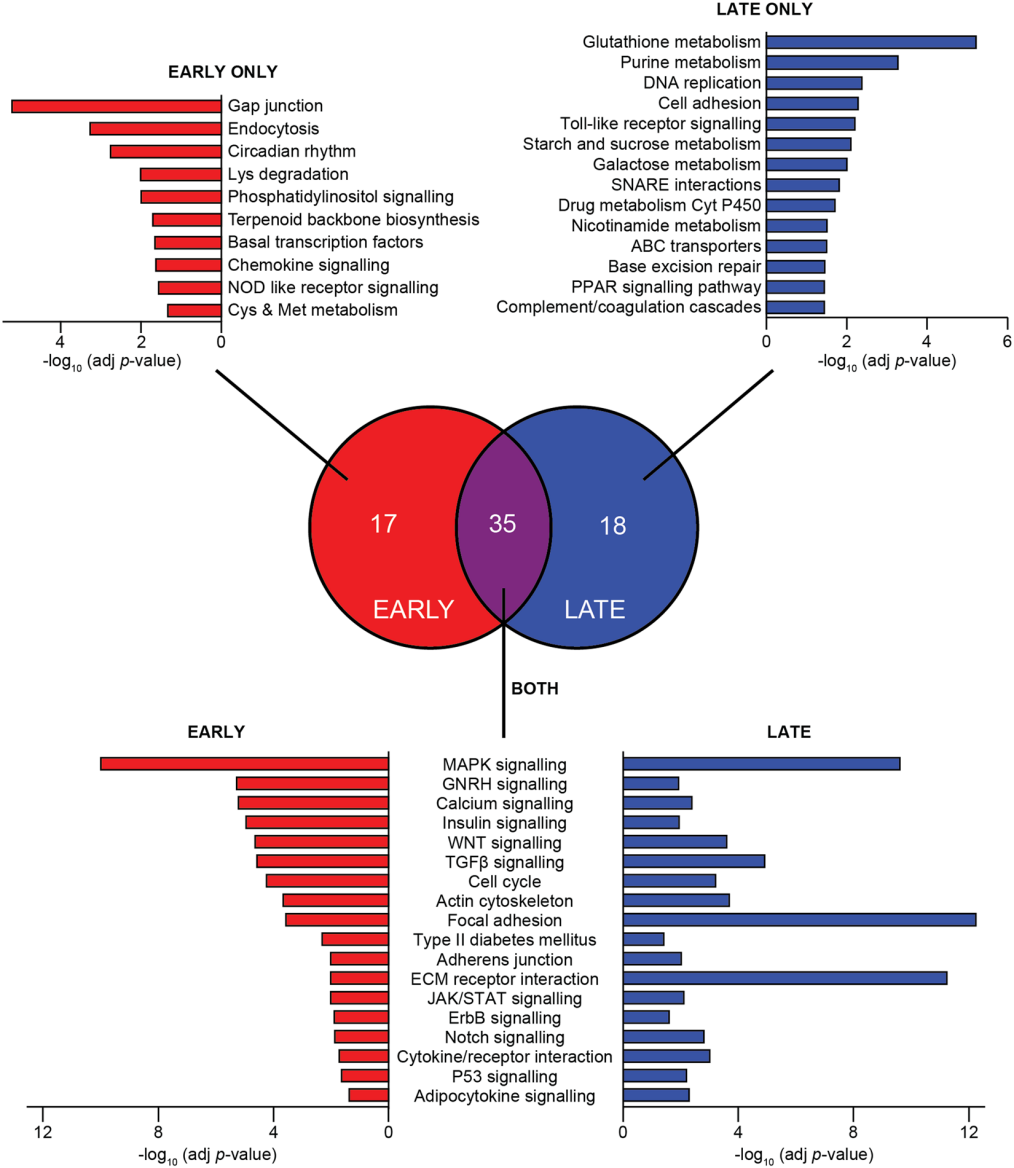
The early and late response to oxidative stress differs in pathway enrichment, but both overlap with insulin resistance. Using the gene expression data from 3T3-L1 adipocytes co-treated with BCNU and auranofin (detailed in Fig. 2), pathway enrichment analysis was performed using genes differentially-expressed either early (2, 4, 8 h) or late (24 h) in the oxidative stress response. Enriched pathways are defined by FDR < 0.05 (details in the Materials and Methods). The Venn diagram depicts the overlap of all enriched pathways, with selected pathways and their adjusted (adj) *p*-values depicted adjacent to the Venn diagram. Enriched pathways with little relevance to adipocyte biology (cancer, neuronal function, cardiomyopathy, and microbial infection) have been omitted for clarity. The complete list of enriched pathways and their associated statistics can be found in Supplementary Table S3. Abbreviations: Cys, cysteine; Met, methionine.

Next, we examined which pathways were transcriptionally regulated under oxidative stress. We considered two time periods in response to BCNU and auranofin co-treatment, early (2–8 h) and late (24 h), for two reasons. First, transcriptional changes occur on the scale of hours^22^. Second, although there were a comparable number of differentially-expressed genes at 8 h (2397 genes) versus 24 h (2023 genes), we hypothesised that the acute and chronic response to oxidative stress would differ. To explore this, we identified pathways that were significantly enriched (adjusted *p*-value < 0.05) amongst genes that were differentially-expressed in these two time periods (Fig. 3, Supplementary Table S3). The primary pathway database that we chose was KEGG^23^ because it is popular, manually-curated, has a high inner-coherence, contains disease-specific annotations, and is academically available in a downloadable format^24,25^. The KEGG pathway ‘PPAR signalling’ was enriched in the late responders (adjusted [adj] p-value = 3.65 × 10^–2^), and ‘Type II Diabetes Mellitus’ (adj *p*-value = 5.00 × 10^−3^ for early, 3.92 × 10^−2^ for late) and ‘insulin signalling’ (adj *p*-value = 1.15 × 10^−5^ for early, 1.16 × 10^−2^ for late) were enriched amongst genes differentially expressed throughout the time-course, reinforcing the link between oxidative stress and insulin resistance.

Furthermore, numerous signalling pathways were altered in both the early and late time periods, whereas metabolic pathways were either specifically regulated at one time-period (Fig. 3, Supplementary Table S3). For instance, amino acid (‘lysine degradation’, adj *p*-value = 9.86 × 10^−3^; ‘cysteine and methionine metabolism’, adj *p*-value = 4.64 × 10^−2^) and lipid (‘terpenoid backbone synthesis’, adj *p*-value = 1.99 × 10^−2^) metabolism gene sets were enriched in the early time-period, whilst sugar metabolism (‘purine metabolism’, adj *p*-value = 5.40 × 10^−4^; ‘starch and sucrose metabolism’, adj *p*-value = 8.00 × 10^−3^; ‘galactose metabolism’, adj *p*-value = 1.00 × 10^−2^) genes were enriched in the late time-period. This implies that the coordinated regulation of gene expression in response to oxidative stress is temporally regulated and reinforces the utility of capturing the dynamics of this response.

**Figure 4:**
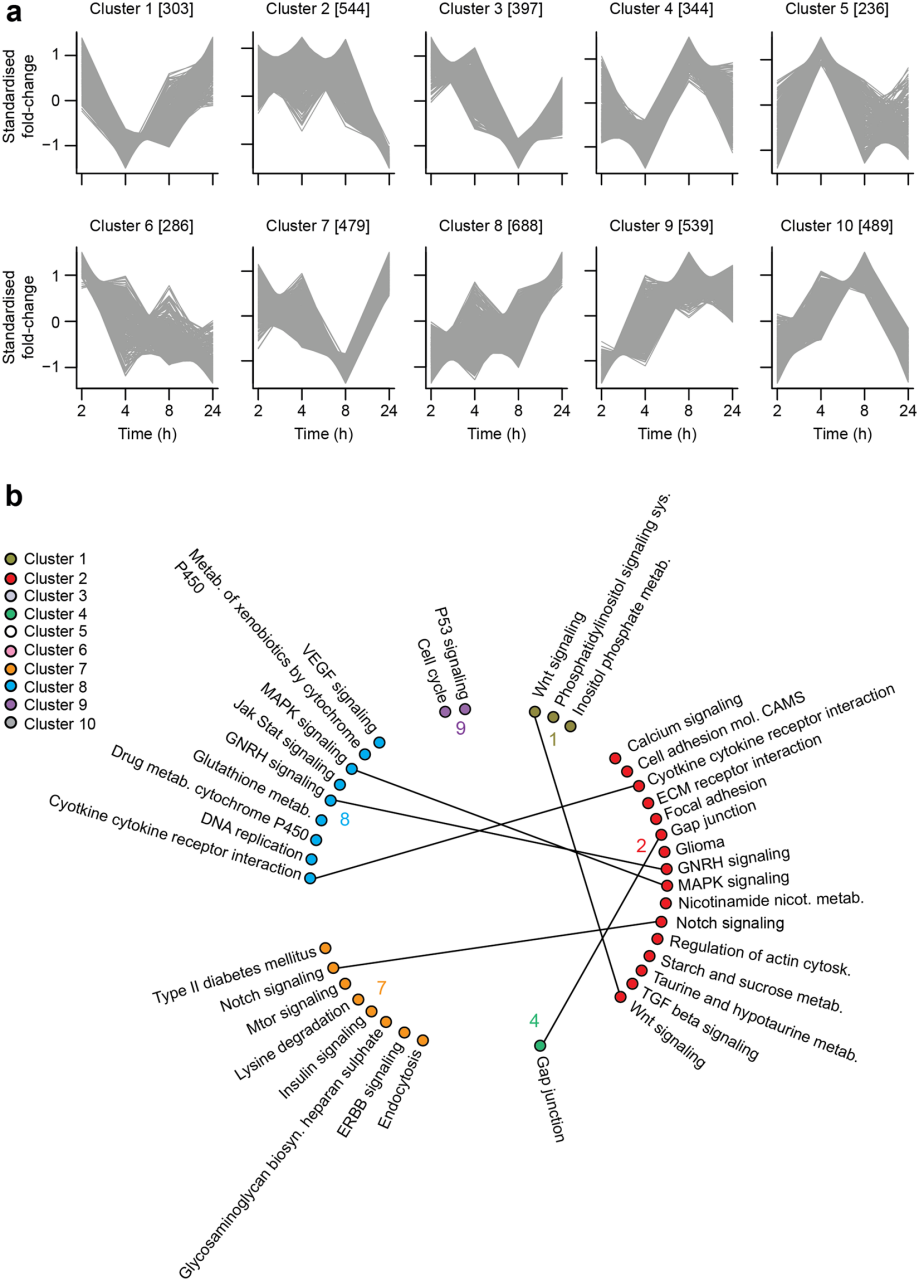
Oxidative stress stimulates a range of gene expression patterns, accompanied with different temporal patterns of pathway enrichment. (**a**) Using the gene expression data from 3T3-L1 adipocytes co-treated with BCNU and auranofin (detailed in Fig. 2), a hierarchical clustering analysis was performed on the differentially-expressed genes using prior knowledge of TF-TG interactions, as described in the Methods and Materials. The parenthesised numbers denote the number of genes in each cluster. Members of each cluster are detailed in Supplementary Table S4. (**b**) Pathway enrichment analysis of each cluster. Only six out of the ten gene clusters enriched for pathways. Selected pathways are depicted, with the complete list and associated statistics found in Supplementary Table S4. Common pathways between clusters are connected by lines.

We next clustered differentially-expressed genes to identify distinct temporal expression patterns in response to oxidative stress (Fig. 4a, Supplementary Table S4). This is typically performed using k-means or fuzzy c-means clustering^16^, but we employed hierarchical clustering here because we obtained the highest enrichment score for optimal cluster selection^17^ (1.0 (K = 10) compared to 0.78 (K = 9) for k-means) and 0.81 (K = 9) for c-means. Furthermore, we augmented the clustering analysis using an approach previously applied to phosphoproteomics data^17^, whereby we optimised the number of clusters using prior knowledge of experimentally-validated TF-TG interactions from the ORTI database^18^. The optimal number of clusters was evaluated based on the correct clustering of known TGs of each TF together, as annotated in the ORTI database^18^. This approach optimally partitioned the data into ten transcriptionally meaningful clusters with distinct temporal profiles (Fig. 4a). The ‘insulin signalling’ pathway was enriched amongst differentially-expressed genes that rapidly declined in expression (Cluster 7, Fig. 4b, adj *p*-value = 3.22 × 10^−3^ for enrichment in Cluster 7), whilst ‘glutathione metabolism’ increased steadily over the time-course (Cluster 8, Fig. 4b, adj *p*-value = 9.53 × 10^−8^ for enrichment in Cluster 8). Interestingly, ‘MAPK signalling’ was also found in Cluster 8 (adj *p*-value = 1.33 × 10^−5^ for enrichment in Cluster 8), enriched by different genes to ‘glutathione metabolism’, suggesting that distinct pathways were co-regulated in response to oxidative stress.

To understand the drivers of this co-regulation, we next determined how TF activity was altered temporally in response to oxidative stress. Using the ORTI database^18^, we identified TFs whose TGs were significantly enriched (*p* < 0.05) in each cluster. 8 out of 10 clusters were enriched for 63 TFs in total (Supplementary Table S4). The directional changes of the clusters account for whether TFs up- or down-regulate their TGs, allowing us to establish connections between TFs and cluster(s) of TGs in a data-driven, unbiased fashion.

**Figure 5:**
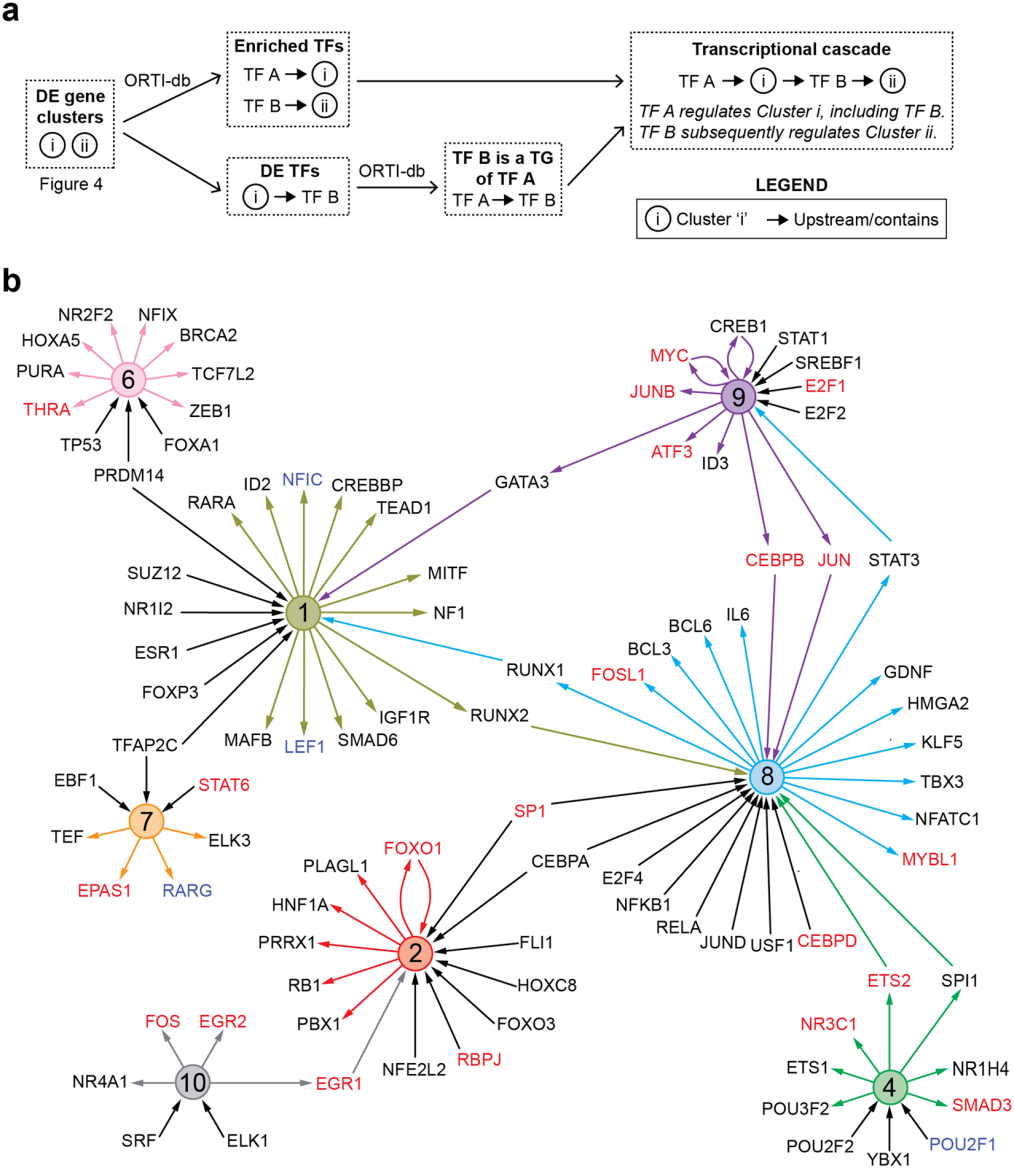
Construction of the transcription cascade triggered in response to oxidative stress. (**a**) Schematic depicting the construction of the cascade. Using the clusters generated in Fig. 4, differentially-expressed transcription factors (TFs) are identified. For each cluster, enriched TFs are determined using the ORTI database^18^, which contains validated target genes (TGs) for each TF. The list of enriched TFs and associated statistics can be found in Supplementary Table S4. In the example, TF A is enriched in Cluster [i], placing TF A upstream of Cluster [i]. TF B is differentially-expressed in Cluster [i], a target gene of TF A, and enriched in Cluster [ii]. This places TF B in between Cluster [i] and [ii]. (**b**) The transcriptional cascade resulting from the steps performed in (**a**). Each coloured circle depicts the cluster number corresponding to the clusters in Fig. 4. Arrows pointing away from cluster circles denote differentially-expressed TFs and arrows pointing towards cluster circles denote TFs enriched in that cluster. If a TF is differentially-expressed in one cluster and enriched in another, its adjacent lines adopted the colour of the cluster in which it was differentially-expressed – this served to highlight TF-cluster interactions that connected two clusters together. TF labels in red font denotes TFs that were transcriptionally upregulated in early adipogenesis^18^, whilst blue font denotes downregulation in expression.

We subsequently constructed a transcriptional cascade, linking two clusters together if a differentially-expressed TF was found in one cluster and its TGs were enriched in another cluster (Fig. 5a). In the example given, TF ‘B’ is differentially-expressed and is itself found in Cluster ‘i’ (Fig. 5a). Furthermore, its TGs are enriched in Cluster ‘ii’. This places TF ‘B’ downstream of Cluster ‘i’ (more specifically, downstream of TFs regulating Cluster ‘i’) and upstream of Cluster ‘ii’ (i.e. regulating Cluster ‘ii’). Thus, Clusters ‘i’ and ‘ii’ are connected by TF ‘B’. In addition, since a transcriptional cascade requires one TF to regulate the expression of another TF, we only included enriched TFs if one of their TGs was also a TF (Fig. 5a). Continuing the example given, TF ‘A’ is enriched in Cluster ‘i’ (in which TF ‘B’ resides) and TF ‘B’ is a known TG of TF ‘A’, together placing TF ‘A’ upstream of TF ‘B’ in the cascade (Fig. 5a).

This approach revealed a complex network of transcriptional interactions in response to oxidative stress (Fig. 5b). Each cluster contained differentially-expressed TFs and was in turn regulated by (enriched) TFs. There were numerous upstream TFs (e.g., ESR1 upstream of Cluster 1, *p*-value = 3.81 × 10^−2^ for enrichment in Cluster 1) that were themselves not found in a cluster, implying their transcriptional activity was likely regulated by other means, such as post-translational modifications (e.g., disulphide bond formation, phosphorylation). Conversely, there were differentially-expressed TFs downstream of clusters (e.g. RARA downstream of Cluster 1) that were not enriched in other clusters. This could be due to their TGs being regulated by other TFs, the lack of information available about their TGs in the ORTI database or changes in their TGs could not be detected under these particular experimental conditions.

### Time-resolved transcriptomics highlights putative TF-TF interactions but requires additional information to fully elucidate transcriptional cascades

**Figure 6:**
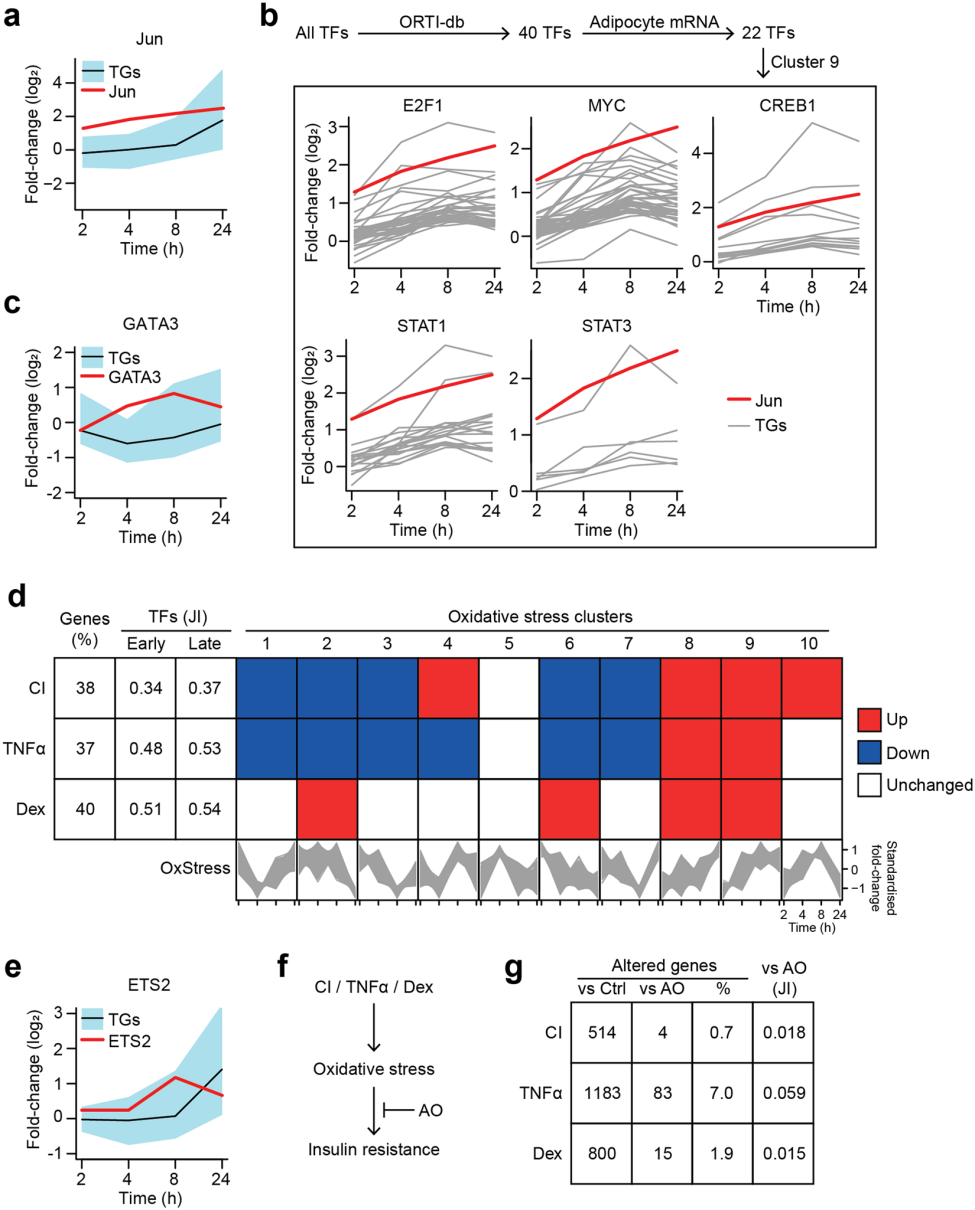
The transcriptional response to oxidative stress shares feature with insulin resistance. (**a**) Expression of Jun (in red) and its TGs in Cluster 8 (defined in Fig. 4): black line depicts the average profile, blue-shading depicts range). (**b**) Schematic depicting the filtering of candidate transcription factors (TFs) that regulate Jun expression. For the 5 potential TFs, their putative target genes (TGs) in Cluster 9 (Fig. 4) are depicted in grey, except Jun in red. (**c**) Expression of GATA3 and its TGs in Cluster 1 depicted as in (**a**). (**d**) The transcriptional response to oxidative stress (described in Fig. 4) was compared to gene expression datasets of several insulin resistance models, including 3T3-L1 adipocytes exposed to chronic insulin (CI) treatment, tumour necrosis factor alpha (TNFα), or dexamethasone (Dex) (Supplementary Table S5). First column displays percentage of differentially-expressed genes in each insulin resistance model that were also differentially-expressed in the oxidative stress time-course (described in Fig. 4). Second/third columns show the Jaccard index (JI, intersection/union) of TFs that were differentially-expressed and/or enriched by oxidative stress (early: 2, 4, 8 h; and late, 24 h) and each insulin resistance model. Subsequent columns display a Gene Set Enrichment analysis, testing the direction of each cluster (Fig. 4) in insulin resistance (details in Supplementary Table S6). In the bottom row, cluster patterns have been reproduced from Fig. 4a for clarity. (**e**) Expression of ETS2 and its TGs in Cluster 8 depicted as in (**a**). (**f**) Hypothetical model, whereby treatment with an anti-oxidant (AO, MnTBAP) reverses insulin resistance. Details in the main text and Supplementary Table S7. (**g**) 3T3-L1 adipocytes were exposed to CI, TNFα, or Dex, with or without AO. First column displays the number of genes differentially-expressed compared to the control (Ctrl)-treated cells, which are not insulin resistant. Second column displays the number of genes reversed in expression by AO. Third column reflects the ratio of genes reversed by AO versus total number of differentially-expressed genes in each insulin resistance model, shown as a percentage. Last column shows the JI of the differentially-expressed genes in insulin resistance with or without AO treatment.

One TF of interest from this cascade was Jun because our analysis linked Jun to many TGs involved in glutathione metabolism in Cluster 8 (GCLM, glutamate-cysteine ligase, modifier subunit; GSS, glutathione synthetase; GSTM1, glutathione S-transferase, mu 1; GSTP1, glutathione S-transferase, pi 1; *p*-value = 1.46 × 10^−4^ for enrichment of Jun TGs in Cluster 8, adj *p*-value = 9.53 × 10^−8^ for enrichment of ‘Glutathione metabolism’ pathway in Cluster 8). The expression of Jun was significantly increased from 2 h onwards in response to co-treatment with BCNU and auranofin (Fig. 6a; adj *p*-values < 8 × 10^−5^ for all time-points, Supplementary Table S1). In contrast, a majority of its TGs did not change expression within the next 6 h (Fig. 6a; only 3.6%, 21.4% and 25.0% of Jun’s TGs in Cluster 8 had adj *p*-values < 0.05 at 2 h, 4 h, and 8 h respectively), increasing only after 24 h (96.4% of Jun’s TGs had adj *p*-value < 0.05). This lag suggests that other regulatory events need to occur to alter its activity, such as protein post-translational modifications. Nevertheless, this would contribute to the enrichment of the glutathione metabolism pathway as a late responder (Fig. 3) and in Cluster 8 (Fig. 4b), and may explain the adaptive response to BCNU treatment in terms of increased glutathione levels (Fig. 1c, *left panel*).

Next, we considered TFs upstream of Jun (Fig. 6b). From the ORTI database, there are 40 TFs that have been experimentally validated to regulate Jun expression across a variety of cellular and experimental contexts. Of these TFs, 22 were identified in the transcriptional cascade (Fig. 5b) and only 5 TFs (E2F1, MYC, CREB1, STAT1 and STAT3) had TGs that matched the expression pattern of Jun in our data (Fig. 6b); i.e. Jun resides in Cluster 9 (Fig. 5b), and only these 5 TFs were enriched in (upstream of) Cluster 9 (*p*-values = 2.50 × 10^−3^, 5.98 × 10^−5^, 4.87 × 10^−2^, 4.44 × 10^−2^ and 4.38 × 10^−2^ respectively). Although the relative contribution of these 5 TFs to Jun expression is unclear, this demonstrates the importance of time-series data and clustering analyses to filter for context-specific TF-TG interactions.

In another example, GATA3 expression rose quickly with oxidative stress before returning to basal levels, with its TGs following a reciprocal pattern (Fig. 6c). GATA3 has been shown to suppress adipogenesis by downregulating the expression of miR183^26^ and directly binding the TFs PPARγ and CEBPα/β to suppress their activity^27,28^. Interestingly, CEBPβ TGs belong to Cluster 8, which rises late in the time-course in parallel to the drop in GATA3 expression, suggesting that GATA3 may regulate CEBPβ in this setting. Since CEBPβ is not a direct TG of GATA3, this example also suggests that the temporal analysis can reveal steps in the transcriptional cascade that may not be mediated solely by one TF regulating the expression of another. This example highlights the need for the incorporation of additional information, such as protein-protein interactions, when studying the dynamics of cascades, even across a single ‘ome.

### Oxidative stress shares common transcriptional features with adipogenesis and insulin resistance

Both Jun and GATA3 have been linked to adipogenesis and insulin resistance. For instance, blocking Jun activity pharmacologically with curcumin reduced adipocyte lipid storage^29^ and blocked adipogenesis^29,30^, yet protected against insulin resistance in adipocytes^31,32^. Thus, we next determined the overlap between oxidative stress and adipogenesis or insulin resistance, by comparison to previously published transcriptomic analyses in 3T3-L1 adipocytes.

Interestingly, roughly half (24/42) of the TFs identified in our previous analysis of early adipogenesis^18^ were found in the oxidative stress transcriptional network (Fig. 5b). This concurs with previous findings that oxidative stress promotes adipocyte differentiation^33–35^. This includes increasing the activity of TF CEBPβ^33^, which appears to be activated as part of Cluster 8 here (Fig. 5b, *p*-value = 3.51 × 10^−2^). This suggests that oxidative stress may influence the activity of TFs involved in transcriptional regulation of adipogenic processes, even in differentiated adipocytes.

We next compared the oxidative stress response to that observed in different adipocyte insulin resistance models (Fig. 6d). We used gene expression datasets from different insulin resistance models, including adipocytes exposed to hyperinsulinaemia (chronic insulin, CI), glucocorticoids (dexamethasone, Dex) or inflammatory signals (tumour necrosis factor alpha, TNFα)^3^ (Supplementary Table S5). These reflect different insults known to induce insulin resistance *in vivo*. Although the differentially-expressed genes and pathways vary greatly between each model (Fazakerley *et al*., manuscript under review), we have shown that oxidative stress is a unifying driver of insulin resistance for each model^3^. Indeed, we found that at the individual gene level, a large portion (~40%) of the genes found to be differentially-expressed in each insulin resistance model were altered under oxidative stress (Fig. 6d, first column). At the TF level, we calculated the Jaccard index (JI) comparing TFs implicated (enriched and/or differentially-expressed) between datasets, and found substantial overlap between the TFs that responded in oxidative stress and the insulin resistance models (Fig. 6d) – this was observed when either the early response (second column, JI ranged from 0.34 to 0.51 across the models) or late response (third column, JI ranged from 0.37 to 0.54 across the models) to oxidative stress was considered.

We next compared the directionality of gene expression changes between oxidative stress and insulin resistance. The insulin resistance datasets contained a single time-point (thus, either genes were up-, down-, or unchanged) whilst our oxidative stress dataset contained a time-course with many non-monotonic expression patterns (Fig. 4). To facilitate this comparison, we tested whether members of the oxidative stress response clusters (Fig. 4) were up- or down-regulated in the insulin resistance models (Supplementary Table S6). Interestingly, Clusters 1, 8, and 9 changed in a similar direction under both insulin resistance and oxidative stress (Fig. 6d; Cluster 1: *p*-value = 2.22 × 10^−3^ for down in CI, 2.33 × 10^−2^ for down in TNFα, unchanged in Dex; Cluster 8: *p*-value = 3.60 × 10^−5^ for up in CI, 3.89 × 10^−8^ for up in TNFα, 1.54 × 10^−2^ for up in Dex; Cluster 9: *p*-value = 3.40 × 10^−8^ for up in CI, 8.01 × 10^−5^ for up in TNFα, 3.28 × 10^−2^ for up in Dex, using the Gene Set Test). These clusters were also highly-connected in the oxidative stress transcriptional cascade (Fig. 5b). This suggests that oxidative stress not only resembles insulin resistance at the phenotypic level (Fig. 1e) and pathway level (Figs 3, 4b), but that the relationship between oxidative stress and insulin resistance can be seen at the transcriptional level with the sharing of common differentially-expressed genes and TFs (Fig. 6d). This implies that oxidative stress may drive some of the transcriptional responses observed in insulin resistant adipocytes.

To explore this notion further, we examined the TF ETS2 as a case-study. ETS2 belongs to the Ets family of TFs, which are commonly studied in the context of cell immortalisation, carcinogenesis, and immunological development^36–38^. Here, ETS2 connects Clusters 4 and 8 (Fig. 6e, *p*-value = 4.08 × 10^−4^ for enrichment in Cluster 8). Its expression increases in response to oxidative stress, with its TGs rising later in the time-course (Fig. 6e). Two of its putative TGs include the TFs FOSL1 (FRA1)^39^ and RUNX1 (AML1)^40,41^, suggesting ETS2 is a key driver in the transcriptional cascade (Fig. 5b). This concurs with previous findings, where ETS2 expression was induced by oxidative stress^42^, sensitising fibroblasts^42^ but protecting glial cells^43^ from ROS-induced apoptosis. In adipocytes, ETS2 has been shown to be essential for adipogenesis^44^. Indeed, ETS2 expression increases under early adipogenesis^18,44,45^ and in every 3T3-L1 insulin resistance model (Fazakerley *et al*., unpublished data). Interestingly, there is also evidence that ETS2 is regulated by insulin-stimulated kinase signalling^46^ and mediates insulin-responsive gene expression^47^. Together, this suggests that ETS2 is utilised by the adipocyte to adapt to a range of stimuli including oxidative stress.

### Transcriptional changes induced by oxidative stress are not sufficient for insulin resistance

Our analyses thus far have shown that oxidative stress triggers a transcriptional response that resembles a substantial portion of the transcriptional changes observed in several insulin resistance models. The next question that arises from this is whether insulin resistance is triggered via oxidative stress *per se* or via the concomitant changes in gene expression that are triggered via oxidative stress. We have previously shown that treatment with an anti-oxidant (Mn(III)tetrakis (4-benzoic acid) porphyrin, MnTBAP)^48^ rescued insulin-responsiveness (translocation of the glucose transporter GLUT4 to the plasma membrane) in each insulin resistance model^3^, demonstrating that oxidative stress is necessary for insulin resistance (Fig. 6f).

Hence, we hypothesised that the transcriptional response to oxidative stress was essential for insulin resistance. We tested this by examining transcriptional changes in each insulin resistance model in response to the anti-oxidant (Supplementary Table S7). Specifically, we looked for genes differentially-expressed versus control-treated cells in each insulin resistance model (Fig. 6g, first column). Then, we assessed whether these genes were differentially-expressed in the reverse direction by anti-oxidant treatment, comparing each model without versus with anti-oxidant treatment (Fig. 6g, second column). Unexpectedly, the anti-oxidant induced very few transcriptional changes (Fig. 6g, third and fourth columns; reversing at most 7% of the transcriptional changes [JI = 0.059] in any insulin resistance model), demonstrating that the anti-oxidant rescues insulin action at the phenotypic level (glucose transport) without reversing the underlying transcriptional changes occurring in the development of insulin resistance. Thus, the transcriptional response to oxidative stress is not sufficient for insulin resistance. This is an intriguing finding as it demonstrates that oxidative stress must have effects in addition to transcriptional changes in the adipocyte to cause insulin resistance. Oxidative stress can alter protein function by the development of disulphide bonds; for instance, disulphide bond formation between residues Cys297 and Cys311 can impair the activity of Akt kinase^49^, a key kinase in insulin signalling. Although we have shown that oxidative stress can cause insulin resistance independently of changes in Akt phosphorylation^3,50^, there is a strong possibility that other post-translational modifications are induced by oxidative stress to cause insulin resistance. This could be explored in future investigations using a combination of redox- and phospho-proteomics.

## Conclusion

Overall, we established an oxidative stress model in adipocytes that induces insulin resistance at the phenotypic level. Previous diet studies in humans and rodents have shown that during insulin resistance, the adipose tissue experiences oxidative stress^1,2^. We have also observed this in mice adipose tissue using PRDX dimerisation (Fazakerley *et al*., manuscript under review), an assay used here to confirm oxidative stress in our BCNU/auranofin model. Our model targets endogenous redox buffering systems to provide a ‘physiological’ origin for the oxidative stress, resulting in impaired insulin-responsive glucose transport in adipocytes. Thus, this complements diet studies by examining the specific response to oxidative stress in isolation of other factors accompanying diet exposure.

Analysing this model in a time-series experiment revealed that the adipocyte manipulates different pathways over time in response to oxidative stress. To uncover the mechanisms driving this response, we started with a repository of validated TF-TG interactions and used a clustering analysis to provide an unbiased means of filtering for context-specific TF-TG interactions. Thus, without any prior knowledge of oxidative stress, we were able to generate a transcriptional cascade that links distinct temporal profiles. Using TF-TG interactions also enabled us to identify direct relationships, rather than secondary effects, in a highly nonlinear transcriptional network. We recognise that this may not uncover every regulatory gene driving the response to oxidative stress, including TFs not well-annotated in the ORTI database and non-TF genes that mediate signalling responses such as kinases. However, we were able to identify known regulators such as ETS2, Jun, and GATA3, and we anticipate that this analysis would improve as current TF-TG repositories expand. Although we sought to primarily use a data-driven approach here, future investigations could complement this with knowledge-driven approaches such as incorporating known responders to oxidative stress in the network analysis.

Furthermore, we found that this transcriptional response shared many similarities with adipogenesis and insulin resistance at the gene, TF, and pathway level. Despite this, treatment with an anti-oxidant, which rescues every insulin resistance model at the phenotypic level, reversed very few transcriptional changes observed under insulin resistance. This disconnect implies that the transcriptional response to oxidative stress is not sufficient for insulin resistance. This suggests that the adipocyte utilises transcriptional cascades to respond to the oxidative stress occurring during the development of insulin resistance, but the primary site(s) by which oxidative stress impairs insulin action are likely to occur post-transcriptionally. Together with our examples highlighting that many TFs are regulated post-transcriptionally, future mechanistic studies into time-resolved responses to cellular perturbations would be strengthened by a ‘trans-omic’ approach^51^.

## Materials and Methods

### Cell culture and treatment

3T3-L1 fibroblasts were passaged and differentiated into adipocytes as described previously^52^, using Dulbecco’s modified Eagle’s medium, supplemented with 10% (v/v) foetal bovine serum and 2 mM GlutaMAX. All cell culture reagents were obtained from Life Technologies (Scoresby, VIC, Australia). Cells were treated with 100 µM 1,3-bis-(2-chloroethyl)−1-nitrosourea (BCNU) or 1 µM auranofin for the indicated time periods. Previously published protocols were used to treat cells with glucose oxidase^50^, as well as generate insulin resistance models, co-treated with or without MnTBAP^3^.

### RNA extraction and microarray analysis

RNA was extracted as described previously^53^, using TRI reagent and 1-bromo-3-chloropropane, both obtained from Sigma-Aldrich (Castle Hill, NSW, Australia), for the oxidative stress samples. The RNeasy protocol (Qiagen, Valencia, CA) was used for the insulin resistance samples. Quantity and quality of total RNA samples was determined using an ND-1000 spectrophotometer (Thermo Fisher Scientific) and Bioanalyzer 2100 (Agilent Technologies, Palo Alto, CA), respectively. RNA with RNA integrity numbers > 8, 260/280 values > 1.8 and 260/230 values > 1.8 were considered acceptable.

The RNA samples were then analysed by microarray analysis. The oxidative stress samples were analysed by the Ramaciotti Centre for Genomics (The University of New South Wales, Sydney, Australia) and the insulin resistant model samples were analysed by Genentech (California, USA). For the oxidative stress samples, labelled cRNA was hybridised to GeneChip Mouse Gene 2.0 Arrays (Thermo Fisher Scientific). For the insulin resistance samples, labelled cRNA was hybridised to GeneChip Mouse Genome 430 2.0 arrays (Thermo Fisher Scientific). Initial data analysis files were generated using the Affymetrix GeneChip Command Console software (Thermo Fisher Scientific).

### Western blotting and PRDX assay

Sample preparation was carried out essentially as previously described^54^. Following treatment, cells were washed thrice with ice-cold PBS that had been pre-treated with 10 µg/mL catalase for 1 h. Cells were incubated with PBS containing 100 mM N-ethylmaleimide for 10 min on ice to modify free cysteine residues. Cells were scraped in PBS containing 1% (w/v) SDS, protease inhibitors and 100 mM N-ethylmaleimide. Samples were centrifuged at 13,000 × g for 10 min at room temperature. Fat was removed, supernatant collected, and protein concentration determined by BCA assay (Thermo Fisher Scientific). Proteins were then resolved by non-reducing SDS-PAGE and immunoblotted as described previously^52^, using antibodies against α-tubulin (from Sigma), PRDX2 (from Abcam), and PRDX3 (from Abfrontier).

### GSSG/GSH assay

GSH/GSSG was measured as described previously^55^, with slight modifications. Adipocytes were lysed in ice-cold TEE buffer (10 mM Tris, 1 mM EDTA, 1 mM EGTA pH 7.4) pre-bubbled with N_2_ gas for ~10 min prior to lysis. Immediately following cell lysis, a portion of the lysate was transferred to a separate tube containing 0.1 mM 1-methyl-2-vinylpyridinium triflate (M2VP), which alkylates all reduced GSH. The M2VP-treated sample was utilised to assess GSSG. Both the original sample and the sample treated with M2VP were subsequently centrifuged (10,000 RPM, 4 °C) and supernatant was used to measure GSH and GSSG. Reaction buffer (100 mM NaH_2_PO_4_, 5 mM EDTA, pH 7.4) for the assay was supplemented with 1 mM 5,5′-Dithiobis(2-nitrobenzoic acid) (DTNB) and 0.1 U/ml glutathione reductase. Following the addition of GSSG standards and sample lysates, the reaction was initiated via 1 mM NADPH. The reduction of DTNB was measured via absorbance (412 nm) each minute for a total of 5 min. GSH and GSSG data were normalized to total protein using the Pierce BCA assay.

### Glucose transport

Cells were serum-starved for 2 h in DMEM supplemented with 0.2% (w/v) bovine serum albumin and 2 mM GlutaMAX (with inhibitors present). This was included as part of the treatment period with BCNU or auranofin (e.g., cells treated for 24 h were treated in the culturing media for 22 h and serum starvation media for 2 h). Following serum starvation, cells were assayed for 2-deoxyglucose (a glucose analog) uptake as described previously^56^, using 100 nM insulin.

### Data curation and analysis

#### Microarray analysis of time course of BCNU and auranofin treatments

CEL files from the microarray experiment on platform Affymetrix MoGene-2_1-st were collected for 41345 transcripts and 32 samples (2 replicates of each of the 4 time points (2 h, 4 h, 8 h and 24 h) for 3 (BCNU, AF, BCNU + AF) models and 2 replicates for controls at each time point. The data were pre-processed (background correction and normalization) using Robust Multi-array Average (RMA) algorithm^57^ implemented in the oligo library from bioconductor^58^ on the pd.mogene.2.1.st library by Carvalho B (2015). Library pd.mogene.2.1.st: Platform Design Info for Affymetrix MoGene-2_1-st. R package (version 3.14.1) was used for annotation. Transcripts that could not be mapped to an approved gene symbol were removed from the dataset, reducing the transcript set to 25293. Probes belonging to multiple genes were collapsed by using the median of the average expression of each probe for that gene. This produced a processed dataset with 24581 unique genes for downstream analysis.

#### Microarray analysis of *in vitro* models of insulin resistance and glucose oxidase treatment

Transcripts in the mouse4302 Affymetrix array were pre-processed (background correction and normalization) using Robust Multi-array Average (RMA) algorithm^57^ implemented in the affyPLM^59,60^ package from bioconductor^58^.

#### Differential expression analysis

All differential expression analysis was performed using the two-sided moderated *t*-test implemented in LIMMA package^61^. Correction for multiple hypothesis testing was conducted using the Benjamini and Hochberg method^62^ to control the false discovery rate (FDR) at 5%. A gene was defined to be differentially expressed (DE) at a given time point if its absolute log_2_(fold change) > 0.5 and adjusted *FDR* < 0.05.

#### Chi-squared test

The statistical significance of differences in proportions of DE genes in samples treated with both vs single drugs or at any timepoint compared to adjacent times were assessed using Chi-squared test whose null hypothesis assumed that the frequency of DE genes was independent of the treatment or time. The Chi-squared test was adjusted for continuity using Yates’s correction^21^. Calculations were performed in the GraphPad online tool for the analysis of contingency tables.

#### Cluster analysis

Genes DE at any time point in the BCNU + auranofin model (5795 genes) were clustered using an in-house modified version of the package clueR^17^. We extended the application of clueR package (originally developed for analysing phosphoproteomics data) to gene expression data and for identifying TF-TG interactions and TF signalling events. To achieve this, we used TF-TG annotations from the ORTI database^18^ as the knowledge-base to evaluate the clusters instead of the default kinase substrate relationships.

We also adapted the clueR package^17^ to incorporate hierarchical clustering (HC). This enabled the study to have singular cluster membership for the genes and reduced membership ambiguity. HC was implemented with Euclidean distance for generating the dissimilarity matrix and the corrected Ward’s method^63^, ward.D2. Our evaluation results with different clustering methods showed that HC produced the highest enrichment scores. The optimal number of clusters (K) was determined using the clustOptimal function of clueR package^17^. The HC tree was cut based on this optimal K, derived from the cluster evaluation package clueR.

#### Enrichment analysis

Enrichment for transcription factors (TF) in each of the 10 clusters was determined through Fisher’s exact test using TF-TG annotations from the ORTI database^18^, ranks 1 and 2 only (468 TFs). TFs that are enriched for a cluster is defined with *p*-value < 0.05.

Biological pathway enrichment analysis is based on the KEGG pathway database from the C2 collection, with an FDR < 0.05. This was performed on genes altered early (3217 genes at either 2, 4, or 8 h) versus those altered late (2203 genes at 24 h), as well as on each individual cluster.

Gene Set enrichment analysis was performed to compare the overlap of DE genes at 2 h and across any time point with the DE genes from the glucose oxidase dataset and members of 15 other signature oxidative stress gene sets that were curated from the Molecular Signature Database^64^ (Supplementary Table S2), with the universe being 41220 approved gene symbols from the Uniprot database (version 30/08/2017). Another Gene Set enrichment analysis was performed to test for the direction (up/down) of regulation of the clusters in insulin resistance using the mean rank Gene Set Test^61^, where by the log(base2)-fold changes of the genes were used as the test statistic.

#### Transcriptional cascade

An in-house R function was used to generate the cascade based on the following rules. For each cluster, significantly enriched TFs were determined as described above. The TGs of enriched TFs were only considered in the cascade if these TGs were also DE in the same cluster in which their TF was enriched. If a TF is enriched in a cluster, this placed the TF upstream of the cluster. A TF was placed in between two clusters (denoted here as I and II) if it was DE in Cluster I, a TG of a TF upstream of Cluster I and enriched in Cluster II. These relationships generated the cascade. Visualization software Cytoscape v3.2.1 and Adobe Illustrator CS6 v 691 was used to generate Fig. 5b.

### Data availability

The datasets generated during and/or analysed during the current study are available in the GEO repository: GSE106270, GSE106271, GSE106324.

## Electronic supplementary material

Supplementary Information

Table S1

Table S2

Table S3

Table S4

Table S5

Table S6

Table S7
