# Supplementary material for: The transcriptional response to oxidative stress is part of, but not sufficient for, insulin resistance in adipocytes
Source: Sci Rep. 2018 Jan 29;8:1774. doi: 10.1038/s41598-018-20104-x (PMC5789081; doi:10.1038/s41598-018-20104-x)
Supplement: Supplementary file 1 — Supplementary Information [file 41598_2018_20104_MOESM1_ESM.pdf]

SUPPLEMENTARY INFORMATION

The transcriptional response to oxidative stress is part of, but not sufficient for, insulin resistance in adipocytes

Rima Chaudhuri[1,2,#], James R. Krycer[1,2,#], Daniel J. Fazakerley[1,2], Kelsey H. Fisher-Wellman[3], Zhiduan Su[1,2], Kyle L. Hoehn[5], Jean Yee Hwa Yang[1,6], Zdenka Kuncic[1,7], Fatemeh Vafaee[5,*], David E. James[1,2,4,*]

[1] Charles Perkins Centre, The University of Sydney, Australia
[2] School of Life and Environmental Sciences, The University of Sydney, Australia
[3] Duke Molecular Physiology Institute, Duke University, Durham, NC 27701 USA
[4] Sydney Medical School, The University of Sydney, Australia
[5] School of Biotechnology and Biomolecular Sciences, The University of New South Wales, Australia
[6] School of Mathematics and Statistics, The University of Sydney, Australia
[7] School of Physics and Australian Institute for Nanoscale Science and Technology, The University of Sydney, Australia
# Joint first authors
* Joint corresponding authors

* Correspondence addressed to: Prof. David E. James, Charles Perkins Centre, The University of Sydney, Sydney, New South Wales 2006, Australia. Tel: +612 8627 1621. E-mail: david.james@sydney.edu.au

Supplementary Table S1. Results from differential expression analysis of the transcriptome under BCNU, AF and BCNU+AF treatments.

Supplementary Table S2. Overlap of oxidative stress gene sets and the differentially expressed genes in BCNU, AF, BCNU+AF and Glucose Oxidase transcriptomics data. Tab 'ALL' shows the overlap of DE genes at any time point (2, 4, 8, or 24 h) in the oxidative stress models '2 h' reports the overlap with genes altered only at 2 h to match the Glucose Oxidase experimental conditions, used as a positive control for oxidative stress. In each tab, the top table presents the overlap as a proportion of the number of genes in each oxidative stress gene set and the bottom table tests the statistical significance of this overlap using hypergeometric enrichment analysis.[63]

Supplementary Table S3. Pathway enrichment analysis through Fisher's exact test for the genes differentially expressed early (2, 4, or 8 h) and late (24 h) respectively in the 'Early' and 'Late' tabs. The overlap of differentially-expressed transcription factors (TFs) and enriched TFs in the oxidative stress data at early and late timepoints with the DE and enriched TFs in the 3 models of IR (CI, TNFα and Dex) are reported in the 'Ox Stress TF overlap with IR' tab.

Supplementary Table S4. Enrichment of pathways and transcription factors (TFs) by the members of each of the ten clusters. Each tab (cl_1 to cl_10) shows the pathway and TF enrichment statistics along with listing the cluster members of each cluster.

Supplementary Table S5. Differential expression analysis results comparing the transcriptomes of adipocytes treated with chronic insulin, TNFa, dexamethasone, and glucose oxidase, compared to control untreated cells.

Supplementary Table S6. Statistics and summary of the gene set tests used to test the direction of regulation of the ten clusters in the 3 models of IR. Tabs 'clusters in CI', 'clusters in TNF' and 'clusters in Dex' reports statistics for the up/down tests for each cluster in CI, TNF and Dex models, respectively. The tab 'cluster summary across 3 models' simplifies and summarizes the results from the other 3 tabs by encoding the direction of change as factor 0,1 and 2 signifying no change, up-regulation and down-regulation, respectively.

Supplementary Table S7. Differential expression analysis results comparing models of IR with and without Mntbap drug treatment to reverse insulin resistance. The tabs 'CI.Mntbap vs CI', 'TNF.Mntbap vs TNF' and 'Dex.Mntbap vs Dex' report the results for CI, TNF and Dex, respectively.

a

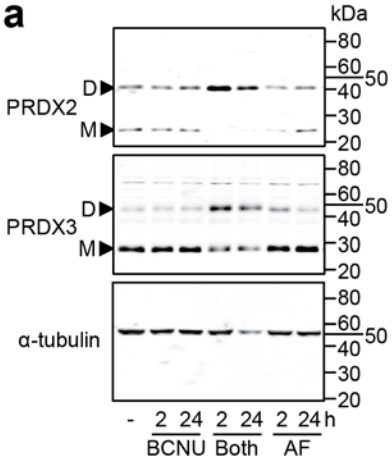

b

		versus control		versus control			Glucose transport	
		PRDX2 (M/D)	PRDX3 (M/D)	GSH (μmol/mg)	GSSG (μmol/mg)	GSH /GSSG	versus control	versus insulin
BCNU	2 h	7.68E-1	1.85E-1	1.44E-5	5.57E-3	6.91E-1	4.32E-2	1.18E-3
	24 h	8.97E-1	1.11E-1	1.06E-2	8.15E-2	1.16E-2	5.46E-1	9.80E-1
AF	2 h	6.98E-2	1.88E-2	9.88E-1	4.64E-1	5.18E-1	2.60E-6	9.81E-1
	24 h	3.25E-1	9.16E-1	1.56E-3	1.29E-6	1.19E-2	4.94E-9	3.38E-3
Both	2 h	2.76E-3	1.70E-3	2.24E-4	2.20E-3	4.06E-2	5.24E-7	1.20E-8
	24 h	2.24E-3	1.07E-3	1.14E-2	6.20E-3	7.62E-1	2.49E-3	6.3E-10
		Figure 1B		Figure 1C			Figure 1D	

$p < 0.05$ (= 5×10^{-2} = 5E-2)
$p < 0.01$ (= 1×10^{-2} = 1E-2)

Supplementary Figure S1. Supplementary data for Figure 1. (a) Full-length blots for the cropped blots depicted in Fig. 1A. (b) *p*-values for the statistical tests performed in Fig. 1.
